# Supplementary material for: Subnuclear organization and mislocalization of plasmids reduce transgene expression
Source: Mol Ther Nucleic Acids. 2025 Oct 9;36(4):102730. doi: 10.1016/j.omtn.2025.102730 (PMC12589873; doi:10.1016/j.omtn.2025.102730)
Supplement: Document S1. Figure S1 and Table S1 [file mmc1.pdf]

OMTN, Volume *36*

Supplemental information

Subnuclear organization and mislocalization of plasmids reduce transgene expression

Ningyang Gu, Uday K. Baliga, Joseph J. Porter, John D. Lueck, and David A. Dean

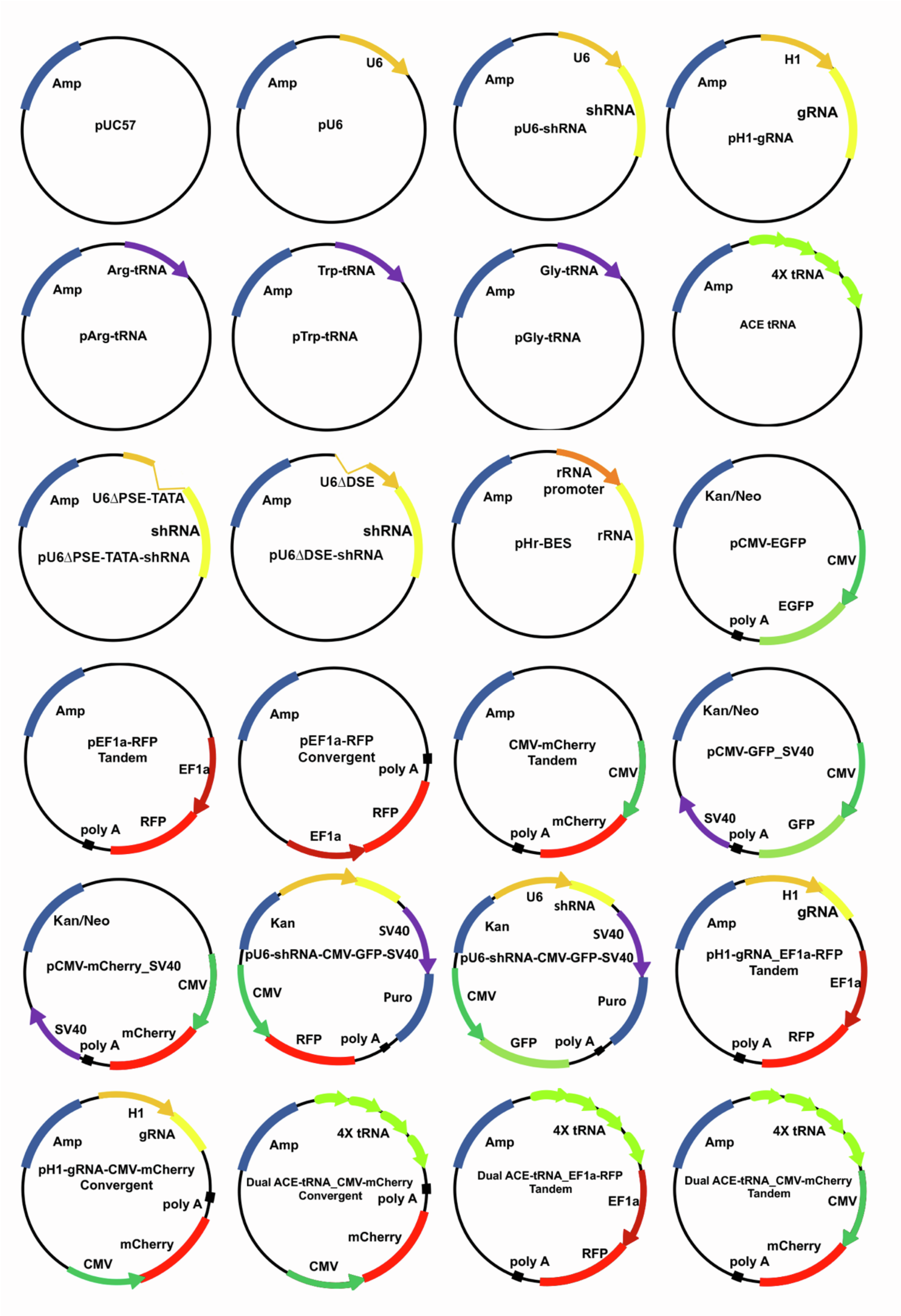

Figure S1. Plasmid Maps

Table S1. Effects of RNA polymerase inhibitors on endogenous Pol II and Pol III expression.

A549 cells were untreated (control), treated with actinomycin D (1 µg/ml) and α-amanitin (5 µg/ml) for 4 hours, or treated with ML-60218 (66 µM) for 24 hours prior to isolation of total RNA. Following reverse transcription, levels of U6 and c-myc were determined by qPCR using the ΔCt/ΔCt method. Primers 5'- CCTGGTGCTCCATGAGGAGAC-3' (c-myc forward), 5'-CAGACTCTGACCTTTTGCCAGG-3' (c-myc reverse), 5'- CGCTTCGGCAGCACATATAC-3' (U6 forward), and 5'- AAAATATGGAACGCTTCACGA-3' (U6 reverse) were used for amplification. Treatments were carried out using n=3-4 wells per condition.

Treatment	c-myc	U6
Control	1.041 ± 0.345	1.007 ± 0.286
1 µg/ml Actinomycin D + 5 µg/ml α-amanitin	0.008 ± 0.004	1.263 ± 0.258
66 µM ML-60218	0.969 ± 0.476	0.633 ± 0.021
