# Supplementary material for: Subnuclear organization and mislocalization of plasmids reduce transgene expression
Source: Mol Ther Nucleic Acids. 2025 Oct 9;36(4):102730. doi: 10.1016/j.omtn.2025.102730 (PMC12589873; doi:10.1016/j.omtn.2025.102730)
Supplement: Document S2. Article plus supplemental information [file mmc2.pdf]

Molecular Therapy
Nucleic Acids
Original Article

Subnuclear organization and mislocalization of plasmids reduce transgene expression

Ningyang Gu,[1,2] Uday K. Baliga,[1] Joseph J. Porter,[3] John D. Lueck,[3,4,5] and David A. Dean[1,3,6]

[1]Division of Neonatology, Department of Pediatrics, University of Rochester, Rochester, NY 14642, USA; [2]Department of Biology, University of Rochester, Rochester, NY 14642, USA; [3]Department of Pharmacology and Physiology, University of Rochester, Rochester, NY 14642, USA; [4]Department of Neurology, University of Rochester, Rochester, NY 14642, USA; [5]Center for RNA Biology, University of Rochester, Rochester, NY 14642, USA; [6]Department of Biomedical Engineering, University of Rochester, Rochester, NY 14642, USA

The expression of DNA within the nucleus is controlled by both promoter structure and sequence and higher-order organization of the nucleus, but how these two affect each other has not been greatly studied. We have previously shown that plasmids carrying Pol II expression cassettes localize to areas of Pol II transcription and processing while those carrying Pol I expression cassettes localize to the nucleolus. Using a microinjection approach in individual cells and imaging both DNA location and gene expression, we find that plasmids carrying Pol III expression cassettes localize in a distinct manner from the other two promoter types in a transcription-dependent manner, similar to all three promoter types. However, when both a Pol II expression cassette and a Pol III expression cassette are carried on the same plasmid, not only does the dual-promoter-class plasmid fail to distribute in a manner similar to plasmids carrying either promoter alone but also expressions of both cassettes are reduced compared to plasmids carrying single-expression cassettes. Taken together, these results confirm that both nuclear organization and promoter structure affect transcriptional outcome.

INTRODUCTION

In transfections and nonviral gene therapy approaches, DNA is delivered to cells with the ultimate goal of having that DNA reach the nucleus in order to be transcribed. The overwhelming assumption is that, once DNA reaches the nucleus, it will be expressed. Consequently, other than optimizing promoter or intron activity, little thought is given to the fate of the DNA inside the nucleus. However, a number of studies show that expressing plasmids display discrete staining patterns, suggesting further movement and/or localization of the DNA once inside the nucleus.[1–9] The subnuclear distribution and movement of genomically encoded genes have been shown to be closely linked to transcription. For example, active transcription of genes can lead to large-scale redistribution of that gene from the nuclear interior to periphery or vice versa.[10–12] While this is also the case for episomally encoded genes, little is known about the impact of subnuclear location on gene expression from these episomes.

In studies using *in situ* hybridization to localize plasmids within the nucleus, it has been shown that the expression cassette carried on the plasmid and transcription itself directs its subnuclear localization.[7,8] Specifically, plasmids expressing either RNA polymerase II (RNAP II) transcripts (mRNA), RNA polymerase I (RNAP I) transcripts (rRNA), or no transcript, all display unique subnuclear organization patterns that are dependent on transcription. When plasmids carrying no eukaryotic promoter sequences were microinjected into nuclei, they remained diffuse and evenly spread throughout the nucleus for at least 4 h post-injection. By contrast, plasmids carrying RNAP II promoter (Pol II) sequences and transcribed mRNA localized to discrete foci within the nucleus starting within minutes and by 4 h were mostly localized to a limited number of areas that colocalized with the splicing machinery.[7] Similarly, plasmids with RNAP I promoter (Pol I) and rRNA coding sequences (CDSs) localized to nucleoli with nucleolar transcription and processing factors.[7,8] In all cases, active transcription was required for this redistribution. However, it is not known whether this redistribution is by simple diffusion and retention or by active transport to specific regions. Perhaps, more importantly, it is also unclear as to what role these subnuclear distribution patterns of exogenously delivered plasmids play in the expression from the plasmids and whether distinct patterns of localization lead to different levels of transgene expression.

In this report, we demonstrate that plasmids carrying Pol III expression cassettes localize to discrete foci within the nucleus that are distinct from those observed for Pol I or Pol II plasmids and that, when multiple promoter classes, such as Pol II and Pol III, are carried *in cis* on the same plasmid, the plasmid redistributes into a completely new pattern unlike those of plasmids carrying single-promoter-type expression cassettes. When expression of either the Pol II (GFP) or Pol III cassette (suppressor tRNA) was measured following injection or transfection of cells, both the total number of transgene-expressing cells and the amount of transgene in each cell were reduced from the dual-promoter-class plasmid compared to single-promoter cassette plasmids. Taken together, these findings

Received 2 June 2025; accepted 1 October 2025;
https://doi.org/10.1016/j.omtn.2025.102730.

Correspondence: David A. Dean, Department of Pediatrics, Box 850, 601 Elmwood Avenue, Rochester, NY 14642, USA.
E-mail: david_dean@urmc.rochester.edu

 © 2025 The Author(s).
Published by Elsevier Inc. on behalf of The American Society of Gene and Cell Therapy.
This is an open access article under the CC BY license (http://creativecommons.org/licenses/by/4.0/).

suggest that alternative approaches may be required for maximizing gene therapy strategies.

RESULTS

Plasmids carrying Pol III type 2 and type 3 promoters do not colocalize with nuclear speckles or nucleoli

To determine the subnuclear distribution and localization of plasmids carrying different promoters, we microinjected ∼5,000 copies of each plasmid (0.25 mg/mL) into the nuclei of cultured A549 human adenocarcinoma cells, and, at indicated times after microinjection, we fixed the cells for fluorescence *in situ* hybridization (FISH) followed by immunofluorescence staining.[2,7] Since it has been reported that anywhere between 2,000 and 100,000 plasmids are taken up by cells during various modes of transfection and that up to 50,000 plasmids have been found to be present in nuclei following lipofection, these numbers of injected plasmids are relevant to gene therapy studies.[13,14] As we have shown previously, when plasmids are visualized immediately after microinjection, they are dispersed relatively evenly throughout the nucleoplasm.[7,8] However, by 4 h, the distribution of injected plasmids shows patterns that are promoter type specific. Since the distribution of the injected DNA within the nucleus is different at 4 h than that immediately following microinjection, we consider this redistribution of the plasmids. We also quantified these qualitative differences in *in situ* hybridization patterns by calculating the percentage of cells that show distribution patterns similar to that shown for a given plasmid. Plasmids carrying the Pol II cytomegalovirus (CMV) immediate-early promoter and enhancer cluster in a number of distinct small areas that colocalize with the splicing factor SC35, a marker for Pol II transcription factories and nuclear speckles, whereas plasmids carrying a Pol I promoter colocalized with nucleolar protein 58 (Nop58, a marker for nucleoli) in the nucleolus (Figures 1A and S1).[7] In all cases, the majority of cells (72.5% ± 5.9%, mean ± SD) injected with a given plasmid show similar redistribution patterns in the nucleus compared to the distribution of the plasmid in the nuclei immediately following microinjection. The percentage of cells sharing the same redistribution pattern is indicated in the overlay panels in each figure.

Since U6 promoter-driven short hairpin RNA (shRNA) is a commonly used Pol III cassette, we tested whether plasmids carrying a Pol III promoter expressing a control scrambled shRNA also redistribute within the nucleus following microinjection. To ensure that the FISH probe only hybridized to injected DNA and not any plasmid-transcribed RNA, we used two different approaches. First, FISH probes were made against the bacterial plasmid sequence, which is not transcribed, and, second, we treated fixed cells with RNase H after hybridization degrade and DNA:RNA hybrids. Four hours after injection, Pol III plasmids redistributed into discrete foci that are spatially different than those of Pol I or Pol II promoter-containing plasmids (Figure 1B). The FISH signal for the Pol III promoter-containing plasmids appears more restricted within small foci compared to the less compact foci or clusters observed for redistributed Pol II promoter-containing plasmids. Using the microinjection approach, 63% of the FISH signal-positive cells showed plasmid redistribution after 4 h compared to the diffuse DNA pattern seen immediately following injection.

To test if Pol III promoter-containing plasmids redistribute into defined nuclear subdomains, we co-stained injected cells after FISH with antibodies against Nop58 and SC35. Nop58 is a key component of the nucleolar ribonucleoprotein complex that assists in the processing of pre-ribosomal RNAs and serves as a marker for nucleoli,[15] and SC35 is a splicing regulator that also regulates transcriptional elongation, mRNA transport, and stability at nuclear speckles. Figure 1C clearly shows that Pol III promoter plasmids do not colocalize with either Nop58 or SC35. Interestingly, removing the shRNA coding sequence completely or generating truncated U6 promoters lacking either the distal sequence element (DES) domain or the proximal sequence element (PSE)/TATA domains still caused the plasmid to localize in subnuclear foci with the same pattern, although with some diffuse DNA staining in addition to discrete foci (Figure 1D).

To test if this localization of U6 promoter plasmids is a phenomenon common among Pol III promoters, we injected an H1 promoter-containing plasmid and anticodon-edited (ACE) tRNA-containing plasmids.[16] The H1 promoter is another commonly used Pol III promoter that is widely used to drive the expression of shRNA and CRISPR guide RNAs (gRNAs), and its efficacy has been evaluated in numerous cell lines. Figure 2A clearly shows that plasmids carrying the H1 promoter also redistribute into small foci that are similar in shape to those seen with U6 promoter-containing plasmids.

While Pol III type 3 promoters such as U6 and H1 contain a TATA box that is upstream of their expressed genes, Pol III type 2 promoters, mainly encoding tRNAs, are intragenic and without TATA boxes, carrying both promoter and transcribed sequence within less than 200 bp. Figure 2B shows that Pol III type 2 plasmids redistribute in a pattern that is similar to Pol III type 3 plasmids despite having different promoter organization and nucleotide sequences. This subnuclear localization appears to occur in multiple cell types, since the U6-shRNA plasmid also formed similar foci in HepG2 cells (Figure 2C). These results suggest that plasmid nuclear redistribution is a common observation rather than Pol III promoter type- or cell type-specific patterns and further suggests that recruitment of transcription complexes or of specific promoter-binding proteins to the plasmid is a key factor directing plasmid nuclear redistribution whereas the downstream CDS may play a lesser role in initiating plasmid redistribution.

A plasmid carrying both Pol II and Pol III promoters exhibits a unique subnuclear distribution unlike plasmids carrying either promoter alone

We demonstrated that plasmids with Pol II promoters colocalize with nuclear speckles, while plasmids with Pol III promoters do not. To examine whether different classes of promoters on the same plasmid affect these redistribution patterns, we injected dual-promoter plasmids that carry a single U6 Pol III and two Pol II

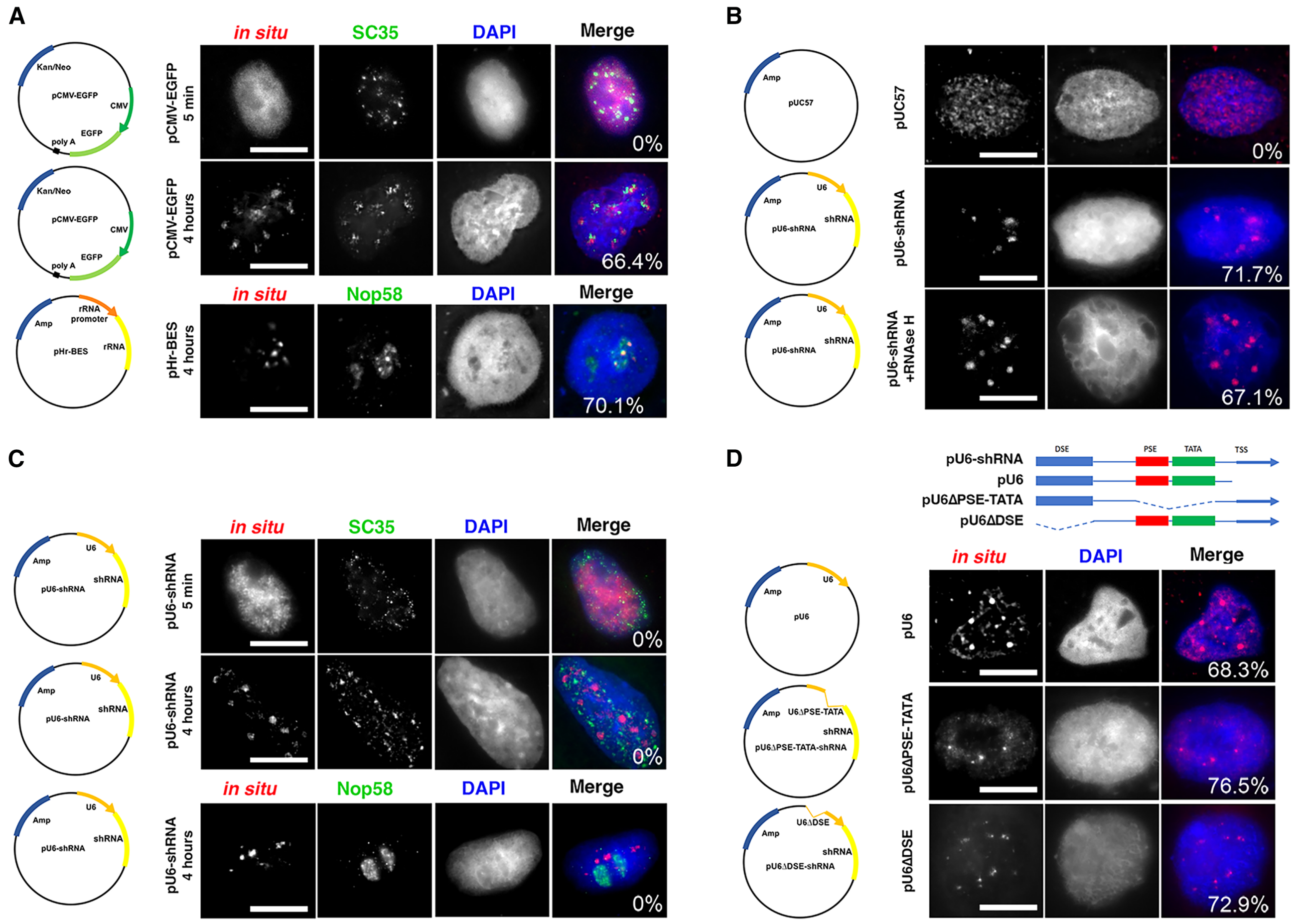

Figure 1. Pol III type 3 promoter-containing plasmids redistribute into small rounded foci that are not coincident with nucleoli or nuclear speckles

(A) Plasmids carrying Pol I or Pol II promoters redistribute and colocalize with nuclear speckles or nucleoli, respectively. The nuclei of A549 cells were injected with pCMV-EGFP (250 ng/μL) or pHr-BES (250 ng/μL) and 5 min or 4 h later processed for FISH using plasmid backbone-specific probes (red), followed by immunofluorescence staining (green) for nuclear speckles (SC35) or nucleoli (Nop58). (B) Plasmids carrying no eukaryotic sequences show no specific redistribution pattern while plasmids carrying a Pol III promoter redistribute into specific foci. A549 nuclei were injected with pUC57 or pU6-shRNA and 4 h later processed for FISH (red). Treatment with RNaseH to digest DNA-RNA hybrids had no effect on the FISH pattern, demonstrating that the FISH signal is indeed that of the plasmids themselves. (C) Pol III promoter-containing plasmids redistribute over time and do not colocalize with previously identified nuclear domains. A549 cells injected with pU6-shRNA were processed 5 min or 4 h after injection for FISH (red) and immunofluorescence staining (green) for nuclear speckles (SC35) or nucleoli (Nop58). (D) Both the Pol III promoter DSE and PSE-TATA are sufficient to cause plasmid nuclear redistribution. Plasmids carrying full-length U6 or truncated U6 promoters shown in the map were microinjected into A549 nuclei and visualized 4 h later by FISH (red). All nuclei are counterstained with DAPI. The percentage of microinjected cells with positive FISH signal that display the shown redistribution patterns of plasmids are indicated for each plasmid on the overlay image. Bar, 10 μm.

promoters (CMViep and SV40 early promoter) directly into the cell nucleus. The SV40 promoter carried on the plasmids in these experiments contains the SV40 early promoter, the ori region, and both 72 bp repeats of the SV40 enhancer. However, since neither A549 nor HepG2 cells express the SV40 T-antigen, the SV40 ori does not support replication in these cells. Given that this U6-shRNA-CMV-GFP-SV40 contains both Pol II and Pol III promoters, we anticipated it would partially colocalize with nuclear speckles. However, this dual-promoter plasmid formed its own distinct plasmid territory within the nucleus unlike either Pol II or Pol III plasmids alone (Figure 3A). This dual plasmid was localized to a large region of nucleoplasm with a clear boundary that does not colocalize with either the nucleolus or nuclear speckles. In fact, the dual plasmid shows no overlap with either nucleoli or nuclear speckles. Nucleolar DNA is enveloped by the dense fibrillar component (DFC) and the granular component, and, after DAPI staining, the nucleolus appears as a region lacking a DAPI signal.[17] Interestingly, the dual-promoter plasmid also localizes to regions that lack a DAPI signal, suggesting that they may possibly share some structural similarities with the nucleolus in terms of being surrounded by proteins that make them inaccessible to DAPI staining.

Next, we investigated whether the unique distribution pattern we observed was limited to specific DNA sequences. We directly

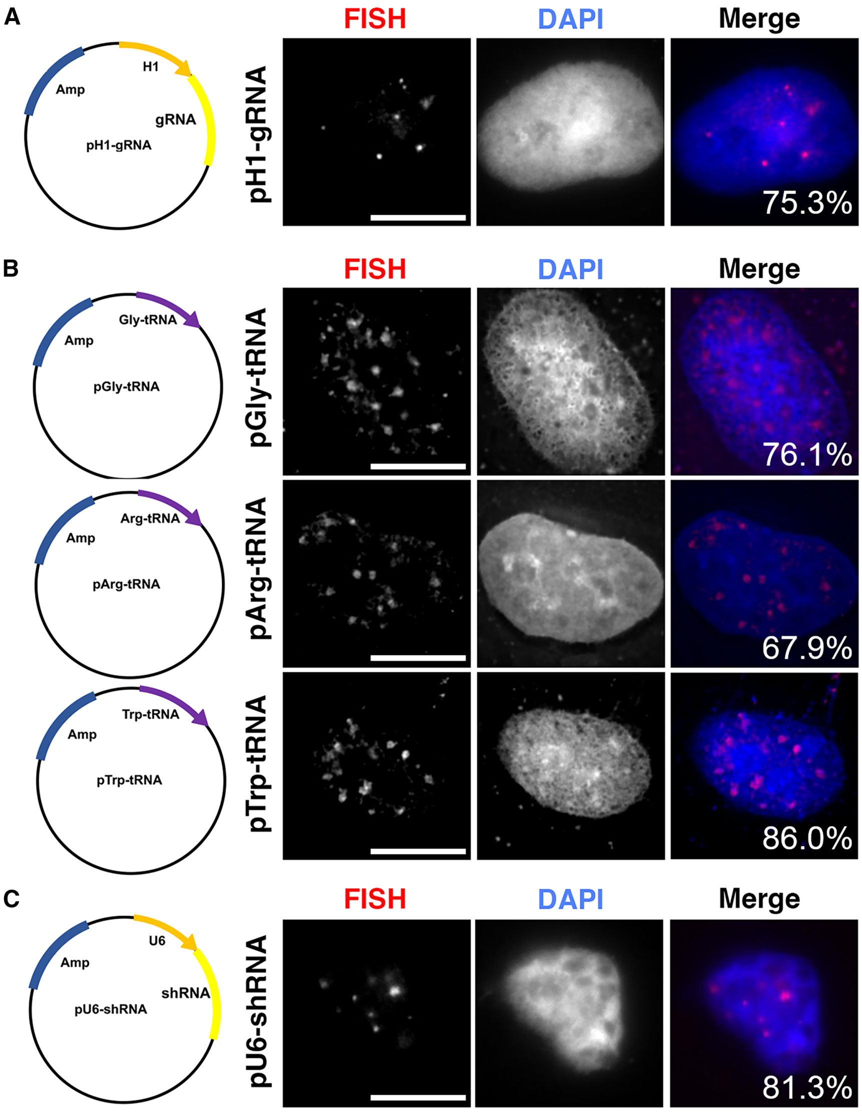

Figure 2. Nuclear redistribution of plasmids carrying Pol III promoters is not promoter type or cell type specific

(A) Plasmids carrying an H1-gRNA cassette redistributed into small foci similar to those seen for U6 promoter plasmids. The nuclei of A549 cells were microinjected with 250 ng/μL plasmid and processed for FISH 4 h later. (B) Pol III type 2 promoters also redistribute into small foci. A549 nuclei were injected with plasmids carrying different ACE-tRNA transgenes, and the distribution patterns were detected 4 h later by FISH. (C) pU6-shRNA injected into the nuclei of HepG2 cells showed nuclear redistribution similar to that seen in A549 cells. All nuclei were counterstained with DAPI. The percentage of microinjected cells with positive FISH signal that display the shown redistribution patterns of plasmids are indicated for each plasmid on the overlay image. Bar, 10 μm.

injected pU6-shRNA-CMV-RFP-SV40, pH1-gRNA-CMV-mCherry, and ACE-tRNA-CMV-mCherry dual-promoter plasmids into the nuclei of A549 cells (Figure S1). Plasmid pU6-shRNA-CMV-RFP-SV40 is similar to U6-shRNA-CMV-GFP-SV40 tested in Figure 3A but contains a CMV-red fluorescent protein (RFP) cassette and a different U6-scrambled shRNA cassette. While the U6 and CMV promoter remain the same, the CDSs of their expression cassettes are different. In Figure 3B, 4 h post-fixation, pU6-shRNA-CMV-RFP-SV40 redistributed into large areas similar to those observed with U6-shRNA-CMV-GFP-SV40, with a distinct absence of DAPI staining. This suggests that the plasmid may have formed its own boundaries or territories.

On the other hand, pH1-gRNA-CMV-mCherry contains a CMV-mCherry expression cassette and an H1-driven gRNA expression cassette. The H1 promoter is generally considered weaker compared to the U6 promoter.[18] We expected pH1-gRNA-CMV-mCherry to distribute more like plasmids containing Pol II promoters, since the H1 promoter is much weaker than the U6 or CMV promoters.[18] However, in Figure 3B, pH1-gRNA-CMV-mCherry still formed a unique pattern, though the lack of DAPI staining in plasmid-injected cells was less obvious than that with pU6-shRNA-CMV-RFP-SV40, and the plasmid did show some degree of co-localization with nuclear speckles. This may suggest that these plasmid territories are less restricted and could have a more rapid transcription factor exchange with the nuclear environment.

We also observed that the dual ACE-tRNA-CMV-mCherry plasmid redistributed similar to Pol III promoter-containing plasmids (Figure 3B). Even though a single Pol III promoter might have weaker transcription factor-binding affinity compared to a Pol II CMV promoter, having 4 Pol III promoters on this plasmid may have shifted the plasmid distribution pattern to small foci. In addition, these foci do not colocalize with SC35, which also implies that the transcriptional activities might be shifted to Pol III promoters. Once again, our data emphasize that promoter strength plays an important role in regulating promoter interactions.

To further support that the influence between different promoters is based on transcriptional activities, pU6-shRNA-CMV-GFP-SV40 was injected after we inhibited either Pol II or Pol III transcription. After inhibiting Pol II transcriptional activity using actinomycin D (1 μg/mL) and α-amanitin (5 μg/mL) 30 min prior to injection, the dual promoter-containing plasmid redistributed into foci that are very similar to those seen with Pol III promoter-containing plasmids.

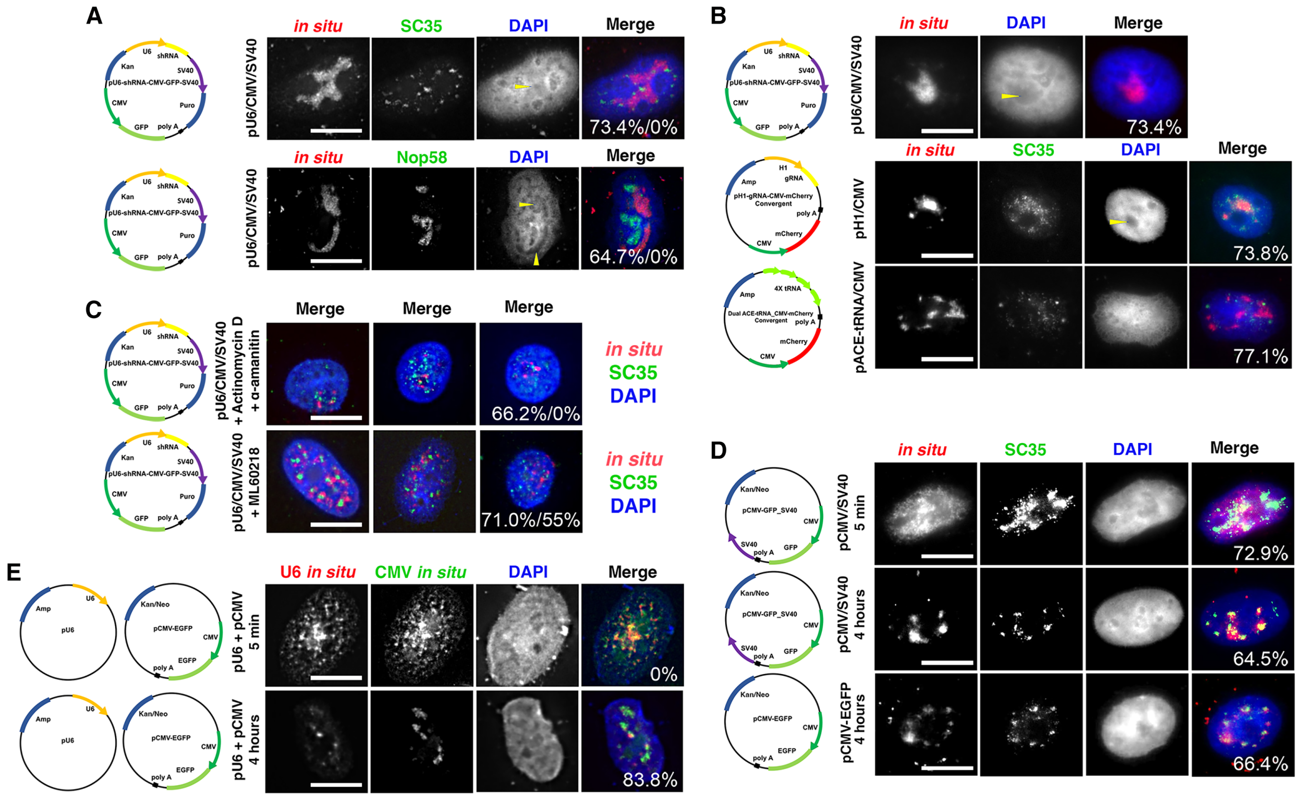

Figure 3. Different types of promoters on the same plasmid affect its nuclear redistribution pattern in a transcription-dependent manner

(A) Plasmids carrying both Pol II and Pol III promoters redistribute into unique patterns. The nuclei of A549 cells were injected with pU6-shRNA-CMV-GFP-SV40 (250 ng/μL). After 4 h, the cells were processed for *in situ* hybridization and immunofluorescence for either Nop58 (nucleoli) or SC35 (nuclear speckles). Yellow arrows indicate areas of restricted DAPI staining. The numbers presented in the overlay image refer to the percentage of injected cells with positive FISH signal that show this redistribution pattern (first number) and the percentage of cells showing colocalization with the stained protein marker (second number). (B) Plasmids carrying different combinations of Pol II and Pol III promoters show similar redistribution patterns. A549 cell nuclei were injected with pU6-shRNA-CMV-RFP-SV40, pH1-gRNA-CMV-mCherry, or pACE-tRNA-CMV-mCherry (250 ng/μL). After 4 h, the cells were processed for *in situ* hybridization and immunofluorescence using FISH probes generated from the plasmid sequences. Yellow arrows indicate areas of restricted DAPI staining. The percentage of microinjected cells with positive FISH signal that display the shown redistribution patterns of plasmids are indicated for each plasmid on the overlay image. (C) Pol II or Pol III transcriptional inhibition resulted in redistribution patterns more similar to plasmids with single promoter types. A549 cells were either untreated or pretreated for 30 min with the Pol II inhibitors actinomycin D (1 μg/mL) and α-amanitin (5 μg/mL) prior to injection or treated with the Pol III inhibitor ML-60218 CAS (66 μM) for 20 h before injection of pU6-shRNA-CMV-GFP-SV40 (250 ng/μL). FISH (red) was performed 4 h after injection, and cells were also stained for SC35 (green). Three representative cells for each treatment are shown. The numbers presented in the overlay image refer to the percentage of injected cells with positive FISH signal that show this redistribution pattern (first number) and the percentage of cells showing colocalization with SC35 (second number). (D) The presence of multiple Pol II promoters on a single plasmid has no effect on redistribution compared to plasmids with a single Pol II promoter. A549 cells were injected with pCMV-GFP-SV40 or pCMV-EGFP (250 ng/μL). After 5 min or 4 h, the cells were processed for FISH (red) and SC35 immunofluorescence (green). The percentage of microinjected cells with positive FISH signal that display the shown redistribution patterns of plasmids are indicated for each plasmid on the overlay image. (E) Injection of a mixture of pU6-shRNA and pCMV-GFP plasmids maintained their Pol II-like and Pol III-like distribution patterns. A549 cells were injected with an equimolar mixture of pU6-shRNA and pCMV-EGFP-SV40 (250 ng/μL). After 5 min or 4 h, the cells were processed for double *in situ* hybridization using red or green FISH probes generated from unique sequences on each plasmid to differentiate the two DNAs (Pol II and Pol III, respectively). The percentage of microinjected cells with positive FISH signal that display the plasmid redistribution patterns are indicated for each plasmid on the overlay image. For each condition, at least 50 cells were injected, and the injections were repeated three times. Bar, 10 μm.

With Pol III transcriptional inhibition using ML-60218 (66 μM) 20 h before the injection,[19] some of the dual promoter-containing plasmids partially restored the Pol II promoter-containing plasmid redistribution and showed some level of colocalization with nuclear speckles, while the rest of the injected DNAs still exhibited a large FISH pattern similar to that seen in the absence of any transcription inhibitor (Figure 3C). Analysis of endogenous gene expression confirmed the predicted specificity of the inhibitors on either Pol II or Pol III transcription (Table S1). This dramatic plasmid distribution change suggests that the promoter-promoter, interaction-induced nuclear redistribution only occurs in a *cis*-manner. Further, the transcriptional activity of one promoter influences the distribution and possibly the activity of the other promoter on the same plasmid.

Next, we injected pCMV-GFP-SV40 directly into the nucleus of A549 cells to determine if the simple property of having two promoters (even of the same promoter type) on a plasmid could alter its subnuclear distribution. Plasmid pCMV-GFP-SV40 contains a CMV-GFP cassette and an SV40 early promoter-driven neomycin resistance cassette. The SV40 early promoter is a relatively strong promoter and is commonly used to drive the selection marker neomycin.[20] At 4 h post-injection, pCMV-GFP-SV40 redistributed into small clusters and colocalized with nuclear speckles (Figure 3D). This suggests that the presence of the same class of promoter does not alter the plasmid's redistribution pattern compared to plasmids carrying only one promoter cassette of the same type.

To test if the same promoter influences would persist when different classes of promoters were carried on separate plasmids (e.g., provided in trans), we injected a mixture of pCMV-GFP-SV40 and pU6-shRNA in equal copy numbers directly into the nuclei of A549 cells. Plasmid pCMV-GFP-V40 contains a CMV-GFP cassette and an SV40-neomycin cassette, while pU6-shRNA contains a complete U6-driven scrambled shRNA cassette and no Pol II promoter. As seen in Figure 3E, at 4 h post-injection, the Pol II promoter-containing plasmid localized to small clusters, while the Pol III promoter-containing plasmid formed rounded foci. Despite being very close to each other, the plasmids showed little colocalization. The Cook lab has shown that even plasmids carrying the same class of promoter form distinct transcriptional active sites, so it is not surprising that Pol II and Pol III promoter plasmids do not colocalize.[9,21] Furthermore, the unique distribution pattern observed when two different promoter types were on the same plasmid (in *cis*), was absent when different single-promoter-class plasmids were mixed in *trans*. Interestingly, a mixture of pU6-shRNA and pCMV-GFP plasmids maintained their Pol II-like and Pol III-like distribution patterns. This suggests that, if the promoters are on different plasmids (in *trans*), they do not influence the localization of the other and provide evidence that the internal chromosome-binding proteins, such as CTCF, likely do not loop the separate exogenous plasmids together. This implies that the plasmids are not incorporated as part of the existing chromosome territories. Indeed, this may be a positive attribute for transgene delivery because it suggests that exogenous plasmids maintain a level of independence from the host genome architecture and function.

Plasmids carrying both Pol II and Pol III promoters exhibit reduced expression of each individual promoters

To investigate how the unique distribution pattern of Pol II-Pol III dual-promoter plasmids affects gene expression, we injected plasmid pCMV-GFP-SV40 and pU6-shRNA-CMV-GFP-SV40 directly into the nucleus of A549 cells, followed by hourly observations of both the percentage of injected cells expressing GFP and the intensity of the GFP signal in each expressing cell. The GFP signal expressed by both plasmids was visible 1 h after nuclear injection (Figure 4A). The dual-promoter plasmid exhibited a slightly but statistically significantly weaker GFP intensity compared to the single Pol II promoter plasmid at both 1 and 2 h, possibly due to plasmid mis-colocalization. After 3–4 h, there was no statistically significant difference in GFP intensity between the dual-promoter plasmid and the single-promoter plasmid. Considering that GFP has a maturation half-time of about 1 hour, its expression should be visible at the early time point.[22] Our results suggest that early transcription is affected by the mislocalization of the dual-promoter plasmid. However, if the produced protein has a longer turnover rate, the total protein level in each cell could eventually even out, leading to similar expression levels over time in individual cells.

After examining the percentage of GFP-positive cells, we observed that cells injected with the dual-promoter plasmid consistently showed fewer GFP-positive cells compared to those injected with the single-promoter plasmid. In Figure 4A, over a 4-h period, approximately 45% of cells injected with the dual-promoter plasmid expressed GFP, while around 65% of cells injected with the single-promoter plasmid expressed GFP. This suggests that, even though the protein level might be similar in individual positive cells, the dual-promoter plasmid has a higher likelihood of silencing the CMV-GFP cassettes after injection and produces less protein across the entire cell population.

To investigate whether the reduced Pol II transcription activity is specific to U6 and CMV promoters or a more general phenomenon, we injected pH1-gRNA-EF1a-RFP and pEF1a-RFP into cells. In Figure 4B, as seen for the U6-CMV dual-promoter plasmid, the H1-EF1a dual-promoter plasmid exhibited weaker RFP expression compared to the single-promoter plasmid at early times post-injection. However, the difference in RFP expression decreased at 3 and 4 h in both H1-EF1a dual plasmid and EF1a plasmid-injected cells but remained statistically significant.

We next tested whether the same was true for type 2 Pol III promoters using a plasmid expressing 4 copies of an ACE-tRNA gene, a plasmid expressing RFP from the EF1a promoter, and a dual promoter carrying both the 4× ACE-tRNA expression cassettes and the EF1a-RFP cassette. For the ACE-tRNA expression cassettes, the endogenous type 2 Pol III promoter was used, with a 55 bp 5′ leader acting as an upstream transcription control element and a short 3′ transcription termination sequence.[16,23] In these experiments, identical copy numbers of plasmids (~5,000) were injected into the nuclei of cells, and the percentage of cells expressing RFP and the levels of RFP in individual cells were measured over time. As seen for the other two combinations of Pol II and Pol III promoters, expression of RFP from the single EF1a-RFP plasmid was higher than that seen from the dual 4× ACE-tRNA-EF1a-RFP plasmid at all times in terms of both the numbers of cells expressing RFP and the intensity of RFP expression in individual cells (Figure 4C).

Subnuclear mislocalization of plasmids affects Pol II and Pol III gene expression in transfected cells

Given that all injections were performed on the same day and on the same coverslip, the injection success rate should not show significant variation within the same set, as we have shown in earlier results.

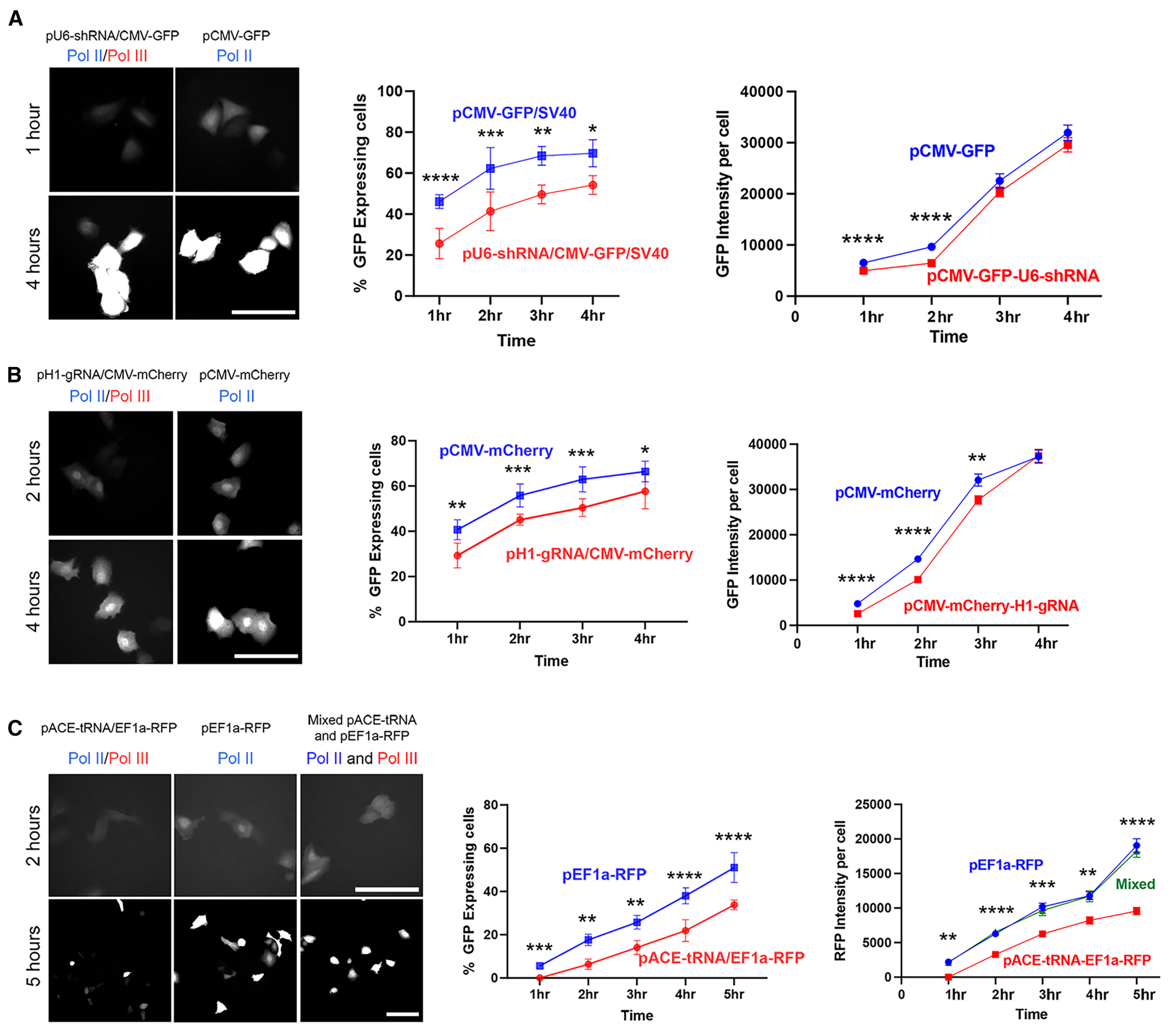

Figure 4. Pol II promoter activity is reduced when a Pol III promoter is present on the same plasmid

The nuclei of A549 cells were injected with either a dual Pol II-Pol III promoter plasmid or the corresponding Pol II plasmid only. Both plasmids were injected on the same coverslip with the same copy number and injection pressure over 15 min to minimize variation. Cells were imaged hourly for GFP expression, and the number of expressing cells was calculated as a percentage of those injected (mean ± SD). Fluorescent protein expression in each expressing cell was determined fluorescence intensity using the ROI function in ImageJ for all injected cells (mean ± SEM). All images used an exposure of 500 ms. The injections were repeated three times, and each time at least 40 cells were injected for each condition. Statistical analyses were by two-way ANOVA followed by Tukey's multiple comparisons tests. *$p < 0.05$; **$p < 0.01$; ***$p < 0.001$; ****$p < 0.0001$. (A) pU6-shRNA-CMV-GFP-SV40 or pCMV-GFP-SV40. (B) pgRNA-CMV-mCherry-Tandem or pCMV-mCherry-Tandem. (C) pACE-tRNA-EF1a-RFP-Tandem or pEF1a-RFP-Tandem. In an additional experiment, a mixture of equal copy numbers of pEF1a-RFP-Tandem and pACE-tRNA plasmids were injected in *trans* and showed similar expression to that seen with pEF1a-RFP-Tandem alone. Bar, 50 μm.

Since dual-promoter plasmids presumably transcribe U6-shRNA and CMV-GFP at the same time, we hypothesized that the reduced GFP transcription could be caused by shifting transcription activities to the Pol III promoter. However, in all of these experiments, we only observed Pol II transcription due to the ability to easily assay for the product of the mRNA produced, namely GFP, by fluorescence microscopy. This does not provide any information on the possible effects of the Pol II cassette on Pol III cassette expression. To follow Pol III expression, we utilized an A549 stable cell line expressing a mNeonGreen fluorescent protein premature termination codon (PTC) suppression reporter transgene. Here, when the PTC is suppressed by the ACE-tRNA, the cells will express functional mNeonGreen, with increasing fluorescence indicating more PTC suppression. Thus, we used mNeonGreen fluorescence as proxy for

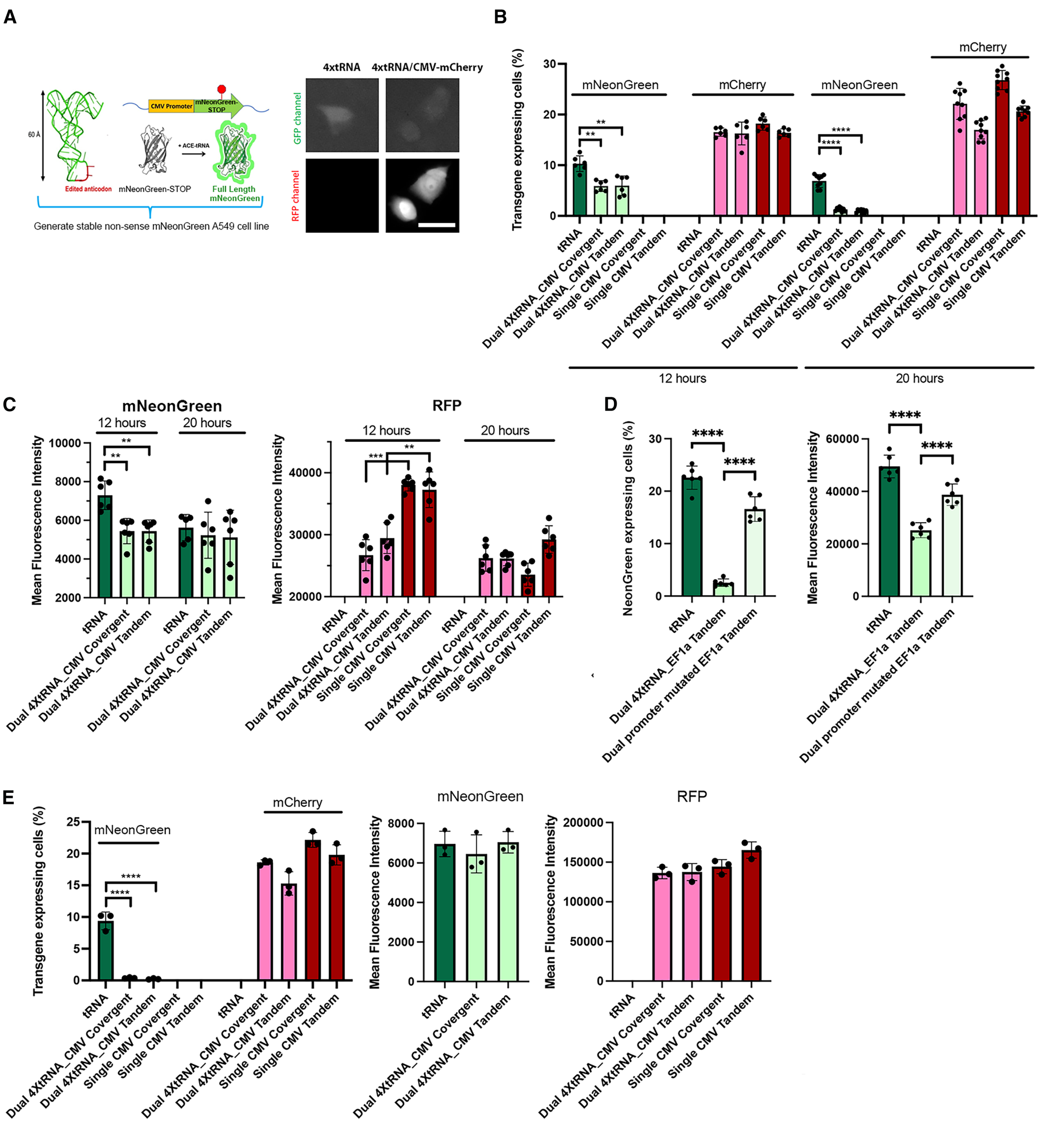

Figure 5. Pol III transcription from plasmids carrying both Pol II and Pol III expression cassettes is severely reduced compared to a plasmid carrying only a Pol III expression cassette in liposome-transfected cells

(A) Use of PTC-reporter mNeonGreen A549 cells and suppressor pACE-tRNA-CMV-mCherry plasmids to quantify Pol III expression. The nuclei of nonsense mNeonGreen A549 cells were injected with either the dual-promoter plasmid p4x ACE-tRNA-CMV-mCherry convergent or a p4x ACE-tRNA plasmid (250 ng/μL). Injections were repeated twice to validate the system, and each time at least 100 cells were injected. After 6 h, the dual p4x tRNA-CMV-mCherry plasmid successfully expressed both mCherry and tRNA-induced mNeonGreen in the same cell, although mNeonGreen expression from the dual plasmid was slightly weaker than that from the p4x ACE-tRNA plasmid alone. (B) The presence of Pol III and Pol II cassettes on the same plasmid reduces the percentage of cells expressing either gene product compared to cells transfected with single cassette plasmids. A549 cells were liposome-transfected with identical copy numbers of either single Pol II or Pol III cassette containing plasmids or with matching plasmids

(legend continued on next page)

Pol III transcriptional activity (Figure 5A).[16] After generating the PTC-reporter mNeonGreen A549 cells, we injected ACE-tRNA-CMV-mCherry-convergent plasmid directly into the cell nucleus to validate the system. Both ACE-tRNA-CMV-mCherry-convergent and ACE-tRNA-CMV-mCherry-tandem plasmids have 4 copies of a Pol III ACE-tRNA expression cassette and one copy of a CMV-mCherry expression cassette. These plasmids carry their Pol II or Pol III expression cassettes either facing each other (convergent) or in the same direction (tandem) to evaluate potential differences in gene orientation in the episomes. As seen in Figure 5A, 5 hours post-injection, PTC-reporter mNeonGreen A549 cells expressed both mCherry and mNeonGreen in the same cell.

Next, we transfected the PTC-reporter mNeonGreen A549 cells with the same copy number of different plasmids and used fluorescence-activated cell sorting (FACS) to measure the expressed mNeonGreen in the population. In Figure 5B, after 12 h, the dual Pol II-Pol III promoter plasmids had fewer mNeonGreen-positive cells compared to the ACE-tRNA only plasmid. Strikingly, this difference increased at the 20-h time point. We hypothesize that this is because the plasmid formed stabilized territories that favor the CMV-mCherry transcription cassette. The ACE-tRNA-expressing positive cells are less than 2% of total cells at 20 h compared to 10% of total cells at 12 h. Interestingly, the mCherry-expressing cells increased slightly at the 20-h time point while ACE-tRNA expression was decreased but did not show a significant difference between single- and dual-promoter plasmids. The significant decrease of ACE-tRNA (i.e., mNeonGreen expression) at 20 h suggests that ACE-tRNA transcription is substantially affected by the presence of the CMV promoter. When examining the mean fluorescence intensity (MFI), we observed a similar trend (Figure 5C): both ACE-tRNA-only and mCherry-only plasmids had higher MFI compared to the dual-promoter plasmid at the 12-h mark. By 20 h, there were no significant differences in MFI, suggesting that early establishment of plasmid redistribution can impact gene expression. Even though the cells with dual-promoter plasmids may accumulate proteins to a certain level and retain biological function, the total number of positive cells in the whole population remains lower for dual-promoter plasmids transfection.

To ensure that Lipofectamine 2000 toxicity did not affect our FACS data, we also balanced each transfection with pUC57, a plasmid containing no eukaryotic promoters, so that all transfected cells received the same total amount of cDNA. As for the previous transfections carried out, cells that were transfected with the 4x ACE-tRNA plasmid in the presence of pUC57 had a much higher percentage of mNeonGreen-positive cells than did the dual-promoter plasmid p4x ACE-tRNA-CMV-mCherry (Figure 5D). However, the RFP signal expressed from the Pol II promoter did not exhibit a significant difference between different groups. At 20 h, the 4x ACE-tRNA-CMV-mCherry dual-promoter plasmid exhibited similar mNeonGreen and RFP intensity as single-promoter plasmids, which could be the result of protein accumulation (Figure 5D).

In comparing the tRNA-only plasmid to the 4x tRNA-EF1a-RFP plasmid, we observed similar results: the percentage of tRNA-positive cells was much lower in cells transfected with the dual-promoter plasmid (Figure 5E). Previous reports have indicated that intron 1 enhances EF1a transcriptional activity and that removal of intron 1 will decrease the Pol II transcription activities.[24–26] Interestingly, after mutating intron 1 (A to G at −327 and −348 relative to the transcriptional start site (TSS) and a deletion at −541), we observed a significant increase in expressed mNeonGreen, indicating increased ACE-tRNA expression. Both the percentage of induced GFP-expressing cells and mNeonGreen MFI increased after mutating intron 1 (Figure 5E). These data suggest that promoter strength can affect the influences between promoters on dual-promoter plasmids and may be crucial for the initial establishment of plasmid territories.

It is well known that the orientation of genes on a plasmid will affect their transcription in bacteria.[27] For instance, in certain experiments, it was found that inserting two genes in a convergent orientation will silence one of the genes significantly. To investigate whether a similar phenomenon occurs in eukaryotic cells, we constructed plasmids by inserting gene cassettes in both convergent and tandem orientations. We found that the orientation of promoters had no significant effect on transcription in these systems and behaved similarly in terms of showing reduced Pol II and Pol III expression compared to single-promoter-class plasmids (Figures 5B–5E). Both convergent and tandem orientation plasmids were capable of expressing both ACE-tRNA cassettes (as assessed by mNeonGreen expression) and CMV-mCherry cassettes. This shows a fundamental difference in the impact of gene orientation on transcription between prokaryotic and eukaryotic systems.

carrying both a Pol II and a Pol III cassette, in either tandem or convergent orientations. Cells were harvested 12 or 20 h later and analyzed by flow cytometry. One-way ANOVA followed by Tukey's multiple comparisons test was used for statistical analysis. $^{**}p < 0.01$, $^{***}p < 0.001$, and $^{****}p < 0.0001$. (C) Mean fluorescence intensity of transfected cells is reduced in cells transfected with dual-promoter-class plasmids. A549 cells were liposome-transfected with either dual-promoter plasmids (convergent and tandem) or single-promoter plasmids (convergent and tandem) using 1 μg of DNA per well and harvested at 12 or 20 h post-transfection for FACS. (D) A transcriptionally active Pol II promoter is needed for reduced expression of the Pol III cassette in plasmids carrying both Pol II and Pol III cassettes. Plasmids carrying only ACE-tRNA cassettes, both ACE-tRNA and a Pol II cassette, or both ACE-tRNA and a Pol II cassette with a mutated promoter were transfected into A549 cells and processed for FACS after 20 h. The mutant EF1a intron plasmid is identical to 4x ACE-tRNA-EF1a-RFP plasmid but carries several mutations (A to G at −327 and −348 TSS and deletion at −541 TSS) in the EF1a intron 1 that prevent RFP expression. (E) The presence of Pol III and Pol II cassettes on the same plasmid reduces the percentage of cells expressing the Pol III gene product compared to cells transfected with single cassette plasmids in balanced mass transfections. A549 cells were liposome-transfected with equal copy numbers of dual-promoter or single-promoter plasmids, and pUC57 was added to balance the mass in all transfections. The cells were harvested at 20 h after the transfection for FACS to determine the percentage of cells expressing mNeonGreen or RFP and the mean fluorescence intensity in productively transfected cells. Bar, 25 μm.

DISCUSSION

In this study, we have shown that Pol III promoter-containing plasmids exhibit distinctive nuclear redistribution patterns. Building upon previous work from our lab and others,[7,8,21] it is clear that Pol II and Pol III plasmids colocalize with their respective transcriptional active domains. Specifically, Pol II plasmids form small, clustered patterns at transcription factories/nuclear speckles,[7,8] while Pol III plasmids (including those with U6 and tRNA promoters) form small rounded foci separate from Pol II domains. These rounded foci are consistent across various Pol III promoters, including H1, U6, and various tRNA genes, despite little similarity in sequence and downstream coding regions. The formation of these rounded foci by Pol III promoter-containing plasmids appears to be a consistent feature and is transcription dependent, suggesting that the recruitment of the transcription complex to the promoter region and/or recruitment of RNA-processing factors to the nascent RNAs transcribed from the plasmids are crucial for plasmid nuclear redistribution. This could imply that the spatial organization of plasmids within the nucleus is heavily influenced by the recruitment of transcription and RNA-processing factors to the promoter region and by newly expressed transcripts from the plasmid.

Unfortunately, we could not identify these foci specifically with antibody staining. Previously, we demonstrated that Pol I promoter plasmids colocalize with nucleoli, a region known for its dense packing of highly repetitive rRNA to avoid possible recombination.[7,8] rRNA transcription occurs in the fibrillar center, while pre-rRNA processing happens in the DFC and ribosome assembly in the granular component.[28] The entire rRNA maturation process is largely confined within the nucleolus. Similarly, Pol II promoter-transcribed mRNA is spliced and matured in nuclear speckles, another distinct nuclear domain.[29] Both rRNA and mRNA are predominantly processed within the nucleus, which might explain why staining for RNA-processing proteins (SC35/Nop58) shows colocalization with the injected plasmids. Unlike mRNA and rRNA, Pol III-transcribed shRNA primarily undergoes processing and maturation in the cytoplasm, not inside the nucleus.[30,31] Only Drosha/DGCR8 cleavage occurs in the nucleus, and the shRNA is rapidly exported to the cytoplasm. This quick export and processing of shRNA could contribute to the rounded foci of Pol III plasmids and further suggests that Pol III transcription may not form a stable nuclear domain like those observed for Pol I and Pol II transcription. Pol III plasmids may remain as small, rounded foci for at least the duration of their expression. Our data suggest that, while the recruitment of transcription factors to the promoter is crucial for initiating nuclear redistribution, the presence of RNA products also facilitates/maintains the distribution process as well.

Our study also revealed that *cis* promoters of different classes on the same plasmid can exhibit a unique nuclear redistribution pattern. Specifically, the U6-shRNA-CMV-GFP-SV40 plasmid formed several large groupings that do not specifically colocalize with nuclear speckles or nucleoli. While it was expected that the plasmid would not colocalize with nucleoli due to the absence of a Pol I promoter, the lack of specific colocalization with nuclear speckles was noteworthy.[7,8] The redistribution pattern of such dual Pol II/Pol III promoter plasmids is not limited to combinations of U6 and CMV but also was observed with H1 and CMV promoter-containing plasmids. Interestingly, the H1/CMV dual-promoter plasmids seem less restricted compared to the U6/CMV promoter plasmid. Even though the H1 promoter is somewhat weaker than U6 promoter,[18] it is still strong enough to cause plasmid redistribution, albeit to a lesser degree. The importance of the promoter strength is further supported by transcriptional inhibition experiments. The directionality of the promoters on the plasmids also does not appear to change the localization patterns of the dual-promoter-class plasmids; both plasmids with Pol II and Pol III expression cassettes facing each other in a convergent orientation and plasmids with tandem Pol II and Pol III expression cassettes in the same direction show the same altered subnuclear distribution. While the distance between Pol II and Pol III promoters in these plasmids varies from 82 to 631 bp, it is possible that increasing the distance between the promoters may mitigate the altered localization and cause the plasmids to distribute to either the Pol II or Pol III domains and increase expression from either or both, but this seems unlikely.

Treatment with a Pol II inhibitor resulted in the U6-shRNA-CMV-GFP dual-promoter plasmid redistributing into rounded foci, a pattern more typical of Pol III promoter-containing plasmids. Conversely, with Pol III inhibition, the same dual-promoter plasmid forms small clusters, reminiscent of Pol II promoter-containing plasmid distributions. Adding Pol II or Pol III inhibitors effectively reduces the transcription from the respective promoters, transforming the dual-promoter plasmid distribution pattern effectively into that seen with a single-promoter plasmid (either Pol II or Pol III active). When one type of transcription is inhibited, the remaining transcriptional activity and RNA products dictate the plasmid distribution pattern. Even though the promoters on the same plasmid can influence each other, the presence of multiple same-class promoters does not necessarily change the overall redistribution pattern. Cook and colleagues have shown that similar active genes can be clustered at a close distance.[9] Thus, it is possible that the promoters are still influencing each other and affecting transcription efficiency but are not detectable by epifluorescence microscopy if there are no dramatic changes. When promoters are on separate plasmids, each maintains a distribution pattern similar to that of a single-promoter plasmid, indicating that the interactions and forces influencing plasmid redistribution are localized and do not extend across separate plasmid molecules. It is possible that the transcript length also may play a role in the subnuclear localization of Pol I, II, and III promoter plasmids. While Pol I plasmids expressing different lengths of an rRNA transcript showed different subnuclear localization patterns, we showed that this was due to the presence of specific processing sites included in the transcripts as opposed to the length of the transcript.

The more profound results in this study are that plasmids carrying both Pol II and Pol III expression cassettes have reduced Pol II and Pol III gene expression compared to plasmids carrying only a single type of expression cassette (e.g., Pol II-Pol II or Pol III-Pol III). We observed that dual-promoter plasmids displayed lower Pol II-driven fluorescent intensity at early time points but reached almost the same level of intensity as single-promoter plasmids at a 4-h time point. Additionally, throughout the experiment's duration, dual-promoter plasmids consistently resulted in fewer positive cells expressing the fluorescent protein. This reduced Pol II gene expression is not just limited to U6-shRNA-CMV-GFP plasmid; a 4x ACE-tRNA-EF1a-RFP plasmid expressed significantly weaker RFP in comparison to the EF1a-RFP-only plasmid. The EF1a promoter is weaker than CMV promoter and could be affected by the Pol III promoters more easily. Our data suggest a correlation between promoter strength and plasmid co-localization within specific nuclear territories, and it is possible that the plasmids that did not express the fluorescent protein from the Pol II promoter might have been actively transcribing Pol III promoter cassettes, which, in the case of the shRNA and gRNAs, could not be detected easily.

To measure the Pol III activity, we utilized the engineered tRNA and nonsense mNeonGreen cell line. What was particularly striking was the dramatic decrease in Pol III transcriptional activity in dual-promoter plasmids. At both 12 and 20 h, there were very few Pol III-positive cells in the dual-promoter plasmid transfections. Even though the MFI of these dual-promoter plasmids might reach levels comparable to that of single-promoter controls at 20-h time point, the percentage of Pol III-positive cells remained lower throughout. Reducing the Pol II promoter activity by mutation also increased the cis-Pol III promoter activity.

Interestingly, in the 4x ACE-tRNA-CMV-mCherry FISH experiment, the dual-promoter plasmid redistributed more like that of a plasmid carrying only a Pol III promoter (Figure 3), and, in the 4x ACE-tRNA-EF1a-RFP injection experiment (Figure 4), the dual-promoter plasmid showed more RFP-positive cells than did cells injected with the RFP plasmid carrying no Pol III cassette. Therefore, we expected that transfection with the 4x ACE-tRNA-CMV-mCherry plasmid would result in lower percentages of RFP-positive cells, compared to the single-promoter plasmids. Interestingly, for both single- and dual-promoter plasmids, there was no significant difference in percentage of RFP (Pol III activity)-positive cells, unlike the percentage of mNeonGreen-positive cells, which is significantly reduced in transfections using the dual-promoter plasmid (compared to the 4x ACE-tRNA-only plasmid). One possible explanation for this could be the trafficking of the cytoplasmic plasmids. In previous studies, we found that a number of transcription factors bind to the SV40 promoter sequence while in the cytoplasm to facilitate the nuclear entry of the plasmids.[32,33] This pre-coupling of transcription factors to the plasmids might drive plasmids to redistribute into Pol II transcription-favored domains as soon as they enter the nucleus, resulting in normal Pol II transcription but a more pronounced decrease of Pol III transcription. Future studies will test this.

Our findings indicate that the dual-promoter plasmid can produce two products at the same time, but with reduced expression from each promoter. Depending on the protein turnover rate, using a mixture of two single-promoter plasmids might be more effective than using a dual-promoter plasmid. Combined with the previous transcription inhibition experiments, our results underscore that mislocalization of the plasmid can decrease gene expression.

Our findings have major implications for plasmid design used in both cell biology and gene therapy applications. Many groups, both academic and commercial, have designed a number of plasmids containing as many "bells and whistles" as possible to make them multifunctional and of maximal utility for multiple applications. This may not be the best approach if maximizing gene expression for an individual gene is desired. For example, it is quite common to include a GFP- or RFP-expression cassette on plasmids that express shRNA to knock down a desired target gene since one can simply assume that the GFP- or RFP-expressing cells have positive knockdown of the shRNA-targeted gene. Our results indicate that maximal expression of either gene cassette is not achieved using this approach; rather maximal levels of transgene expression may be obtained using co-transfection of two separate plasmids, one of which carries a Pol II expression cassette and the other a Pol III cassette. Similarly, expression of a gRNA in a plasmid also carrying a Pol II-expressed product (such as a CRISPR component) may not actually provide greater levels of either product compared to using two separate plasmids. This may be yet another example, albeit biological, of the "less is more" design concept.

With our findings, we propose a model to account for plasmid redistribution within the nucleus. Transcription complexes bind to the promoter region, forming an initial "seed." This seeding is particularly influenced by promoter strength, with stronger promoters more readily forming the initial seed. For plasmids carrying multiple classes of promoters, this initial seeding will depend on the relative strengths of each promoter. Once transcription begins, the newly transcribed RNA and associated RNA-processing proteins contribute to stabilizing or maintaining the plasmid's redistribution pattern, as has been seen with Pol I plasmids, which localize within the nucleus according to the presence of processing sites within the expressed rRNA transcripts.[7,8] These could be small clusters or rounded foci, depending on the specific dynamics of the transcription and the nature of the RNA produced. As protein-RNA/DNA interactions accumulate and reach a critical concentration, these small groupings may fuse together to form more defined "plasmid territories." At this stage, the fate of the plasmid's spatial organization and, consequently, its transcriptional activity is largely determined. For example, this model suggests that, if dual-promoter plasmid forms territories that favor Pol II transcription, the plasmid is less likely to exhibit high Pol III activities, or vice versa. This model

also suggests that the spatial organization of plasmids within the nucleus is a dynamic process influenced by the interplay of transcription factors, promoter strength, and RNA dynamics. Using plasmids that contain multiple promoters will have a reduced expression from each promoter. This may be acceptable when the promoter is strong enough to produce saturated levels of proteins. However, we might encounter some situations where maximized promoter activities become crucial. One aspect of this model and our findings is that it is not known whether the redistribution of plasmids observed is by simple diffusion and retention or by active transport to specific regions. Since our studies used *in situ* hybridization, they show only single time points for any injected cell. Future studies looking at real time movement and distribution of fluorescently labeled plasmids could distinguish between these mechanisms for nuclear organization. However, even without this temporal analysis, our model provides a framework for understanding the complex dynamics of plasmid behavior in the nucleus, offering insights that could be pivotal for enhancing gene therapy techniques, studying gene regulation, and understanding nuclear architecture.

MATERIALS AND METHODS

Plasmids

Plasmid pCMV-GFP-SV40 (pEGFP-C1) expresses GFP from the CMV immediate-early promoter and enhancer and expresses the neomycin resistance gene from the SV40 early promoter (Clontech).[2] pU6 (pSilencer1.0-U6) has one U6 promoter followed by a multiple cloning site but no CDSs (Ambion, Austin, TX). Upon cloning shRNA into the multiple cloning site, this plasmid can be used for shRNA expression. pU6-shRNA expresses a scrambled shRNA cassette from the U6 promoter. It was created by inserting a scrambled shRNA (5′-GCACTACCAGAGCTAACTCAGATAGTACT-3′) downstream of the U6 promoter in pU6. The scrambled shRNA was cloned from pGFP-V-RS-shRNA vector scramble control (OriGene, Rockville, MD). pU6ΔPSE-TATA contains a DSE sequence from the U6 promoter and a downstream scrambled shRNA CDS. It was built by replacing the complete U6 promoter (from pU6-shRNA) with the DSE sequence only. The DSE sequence was amplified from the pU6-shRNA complete U6 promoter with primers (5′-CAAAACGCACCACGTGAC-3′ and 5′-GTCCATTTTAAAA CATAATTTTAAAACTGC-3′). The PSE-TATA box sequence was removed from the U6 promoter after PCR. Plasmid pU6ΔDSE contains a PSE-TATA box sequence from the U6 promoter and a downstream scrambled shRNA CDS. It was built by replacing the complete U6 promoter (from pU6-shRNA) with PSE-TATA box sequence. The PSE-TATA box sequence was amplified from the complete U6 promoter with primers (5′-GCAGTTTTAAAATTAT GTTTTAAAATGGAC-3′ and 5′-TTAGCTCTGGTAGTGCCG-3′). The DSE sequence was removed from the U6 promoter after the PCR. Plasmids ACE-Arg-tRNA, ACE-Trp-tRNA, and ACE-Gly-tRNA all have Pol III type 2 promoter-driven tRNA cassettes cloned into a pUC57 backbone.[16] pU6-shRNA-CMV-GFP-SV40 (pGFP-V-RS-scramble) has a CMV-GFP cassette, a U6-scrambled shRNA cassette, and an SV40-neomycin cassette (OriGene, Rockville, MD). pU6-shRNA-CMV-RFP-SV40 (pRFP-C-RS-scramble) was purchased from OriGene, Rockville, MD. pH1v1 has a complete H1 promoter-driven gRNA cassette (Addgene).[34] pCMV-mCherry-SV40-neo has two Pol II cassettes: one expressing mCherry from the CMV immediate-early promoter and enhancer and the other expressing neomycin resistance from the SV40 early promoter and enhancer (Takara Bio #632524, Kusatsu, Shiga, Japan). pH1-gRNA-CMV-mCherry-convergent has a CMV-mCherry cassette and an H1-driven gRNA cassette. It was built by inserting a CMV-mCherry cassette from pCMV-mCherry-SV40-neo downstream of the H1-driven gRNA cassette in pH1-gRNA. The CMV-mCherry cassette was amplified with PCR (forward: 5′-GTAACCGGTTTCAT GACCCTGATTCTGTGGATAACCGT-3′ and reverse: 5′-GTAAC CGGTTTCATGACATTGATGAGTTTGGACAAACCAC-3′) and inserted on the pH1-gRNA PciI site. pH1-gRNA-CMV-mCherry-convergent contains the two transcription cassettes in a convergent orientation. pH1-gRNA-H1-RFP has an H1-RFP cassette and an H1-driven gRNA cassette. It was built by inserting an H1-RFP cassette downstream of the H1-driven gRNA cassette in pH1-gRNA. Plasmids p4x ACE-tRNA-EF1a-RFP-convergent and p4x ACE-tRNA-EF1a-RFP-tandem have 4 copies of a Pol III ACE-tRNA cassette and one copy of EF1a-RFP with the Pol II and Pol III cassettes in a convergent or tandem orientation, respectively. Plasmid p4x ACE-tRNA-CMV-mCherry-convergent and p4x ACE-tRNA-CMV-mCherry-tandem have 4 copies of Pol III tRNA cassette and one copy of CMV-mCherry cassette, placed in a convergent or tandem orientation, respectively, relative to each other. These two plasmids were constructed by inserting a CMV-mCherry cassette downstream of the 4x ACE-tRNA cassette of plasmid p4x ACE-tRNA. The CMV-mCherry fragment was cloned from pCMV-mCherry-SV40. The CMV-mCherry cassette was amplified by PCR (forward: 5′-GTAACCGGTTTCATGACCCTGATTCTGTGGATA ACCGT-3′ and reverse: 5′-GTAACCGGTTTCATGACATTGATG AGTTTGGACAAACCAC-3′) and inserted on the p4x ACE-tRNA PciI site. p4x ACE-tRNA-EF1a-RFP contains several mutations in EF1a intron 1 region (A to G at −327 and −348 relative to the TSS and a deletion at −541). The Pol II and Pol III cassettes are in convergent orientation, relative to one another. Plasmid pEF1a-RFP has one copy of EF1a-RFP and no Pol III cassettes. It was constructed by removing the H1-gRNA cassette from the pH1-gRNA-EF1a-RFP. Plasmid maps are shown in Figure S1. All constructs were verified by DNA sequencing and prepared using QIAGEN Maxiprep kits (QIAGEN, Chatsworth, CA) and suspended in 10 mM Tris, pH 8.0, and 1 mM EDTA.

Cells and cell culture

A549 human adenocarcinoma cells (ATCC, Manassas, VA) and HepG2 human hepatocellular carcinoma cells (ATCC) were grown in high-glucose DMEM supplemented with 10% fetal bovine serum, kanamycin, and antibiotic/antimycotic solution (Thermo Fisher Scientific, Waltham, MA). After reaching 80%–90% confluency, the cells were detached from the tissue culture plate with trypsin and plated on etched coverslips in a 6-well plate and grown in a humidified 37°C incubator with 5% CO_2 for 24 h prior to microinjection.

An A549 cell line stably expressing NeonGreen containing a nonsense mutation at residue 162 was created by co-transfecting cells with a piggyBac (PB) transposon vector carrying the nonsense NeonGreen gene and the PB transposase vector (C15). Stable cells were selected with 0.5 μg/mL of puromycin (InvivoGen, #ant-pr-1) for 1–2 days post-transfection until no viable cells were detected in the negative control condition. Stable colonies were then cloned and expanded and grown in Gibco DMEM cell culture media supplemented with 10% fetal bovine serum and antibiotics (Thermo Fisher Scientific, Waltham, MA). After reaching 80%–90% confluency, the cells were detached from the tissue culture plate and plated on 6-well plates at a concentration of 50,000 cells per well. When each well reached 80% confluency, cells were transfected using Lipofectamine 2000 in the absence of puromycin.

Transcription inhibition

For Pol II transcription inhibition, cells were treated with actinomycin D (1 μg/mL; Sigma-Aldrich, St. Louis, MO) and α-amanitin (5 μg/mL; Sigma-Aldrich) 30 min prior to injection. For Pol III transcription inhibition, the cells were treated with ML-60218 (CAS no: 577784-91-9; 66 μM; EMD Millipore) 20 h before the injection.[35] Both inhibitors remained present in the media during injections and cell growth until the cells were fixed at the end of the experiment.

Microinjection

Plasmids were diluted to 0.25 mg/mL in 0.5X PBS (68.5 mM NaCl, 1.35 mM KCl, 5 mM Na_2HPO_4, and 0.9 mM KH_2PO_4) and filtered through a 0.22 μm membrane Spin-X tube (Corning-Costar, Cambridge, MA) to remove any precipitates or aggregates. Borosilicate microinjection needles were pulled on a Sutter P-97 needle puller (Sutter Instrument, Novato, CA) and backloaded with plasmid immediately prior to injection using an Eppendorf FemtoJet microinjection system outfitted on an inverted Leica epifluorescence microscope enclosed in an environmental chamber to maintain a 37°C temperature. Plasmids were injected directly into the nuclei with an inject pressure of 150 hPa for 0.3 s. Approximately 60–80 cells were microinjected for each set of experiments, and all sets were repeated at least 3 times. After injection, the coverslips were incubated at 37°C before either fixation for *in situ* hybridization or live imaging.

Plasmid FISH

At the indicated endpoints, coverslips are washed with 1X PBS for 10 s, permeabilized with 1X PBS containing 0.5% Triton X-100 x for 45 s, fixed in 1:1 methanol: acetone for 5 min, and placed in 70% ethanol at 4°C overnight or until further processing. Fluorescent DNA probes were generated by nick translation, and FISH was performed as previously described.[1] To detect double-stranded plasmid DNA, cellular DNA was denatured by incubating the coverslips in 70% formamide in 2X saline sodium citrate (SSC) for 10 min at 70°C, a treatment that leads to denaturation of any fluorescent protein expressed in the cells. Probes were generated from the unique sequences on each plasmid so that multiple different plasmids could be simultaneously visualized within an injected cell. After hybridization, coverslips were washed for 30 min each at 37°C with 50% formamide/2X SSC, 2X SSC, 1X SSC, and finally 1X PBS prior to staining of the nuclei with DAPI and mounting with anti-fade reagent. To determine the percentage of cells that show redistribution of the injected plasmids compared to the distribution pattern of the plasmid seen immediately following nuclear injection (sets of injected cells were fixed within 5 min of injection for this time point), we counted the numbers of cells with DNA signal showing the same pattern of foci. In all cases, the majority of cells injected with a given plasmid showed similar patterns of subnuclear distribution.

Immunofluorescence staining

When required, coverslips were processed for immunofluorescence after FISH. Immediately after the 1X SSC wash, coverslips were washed with 1% BSA in 1X PBS three times and blocked with 1% BSA at room temperature for 1 h. Coverslips were then incubated with primary antibodies (SC35 1:200, Nop58 1:200, Brf1 1:50, TFIIIC 1:50, and SNAPC 1:50) at room temperature for 2 h. Coverslips were washed and incubated with secondary Alexa Fluor 488 antibodies (1:1,000) for 1 h. After this incubation, coverslips were washed 3 times with 1X PBS for 30 min each and stained with DAPI prior to mounting with anti-fade reagent. The following antibodies were used: mouse anti-SC35 (#556363, lot 18802, BD Pharmingen) and rabbit anti-Nop58 (NBP1-81680, lot R09620, Novus Biologicals), rabbit anti-BRF1 (Novus Biologicals, #NBP2-55335, lot #R67284), rabbit anti-TFIIIC (Novus Biologicals, # NBP2-14077, lot #R66983), and rabbit anti-SNAPC1 (Invitrogen, #PA5-84742, lot #UD2748777B).

Microscopy

Fixed cells were observed under a Leica DMRXA epifluorescence microscope with a 100X objective (NA 1.47; Leica, Wetzlar, Germany). Images were taken with a Hamamatsu OCRA-ER CCD camera (Hamamatsu, Japan) and processed with ImageJ Deconvolution Lab plugin. For live-cell imaging, after microinjection, cells were imaged with a 40X objective every hour for a total of 4–6 h using a Hamamatsu OCRA-ER CCD camera (Hamamatsu, Japan).

Transfection and flow cytometric analysis

Lipofectamine 2000 was used as described by the manufacturer. All plasmids were transfected at the same copy number based on their size. Twelve or 20 h after transfection, cells were detached from the plates with trypsin, spun down at 3,000 rpm, and resuspended in 300 μL PBS. Debris was removed by filtration with 5mL polystyrene round-bottom tubes with cell-strainer caps, and tubes were stored on ice and processed within 4 h for flow cytometry using a 4-laser (UV-V-B-R) Cytek Aurora spectral flow cytometer (Cytek Biosciences, Fremont, CA). Unmixing parameters and gating were determined using single positive controls. Collected data were analyzed using FCS Express (De Novo Software, Pasadena, CA). Fluorescent intensity measurements were carried out by comparing the geometric MFI of the noted population for mCherry, NeonGreen, or both.

DATA AND CODE AVAILABILITY

All experimental data are available upon request to the corresponding author.

ACKNOWLEDGMENTS

This work was supported by grants GM151450 (D.A.D.), EB009903 (D.A.D.), and HL153988 (J.D.L.) from the NIH and a grant from the Cystic Fibrosis Foundation (000541256-SC001-Lue, J.D.L.).

AUTHOR CONTRIBUTIONS

N.G., investigation (lead), formal analysis (lead), visualization (lead), writing – original draft (equal), methodology (supporting), and writing – review and editing (supporting). U.K.B., methodology (supporting), investigation (supporting), formal analysis (equal), and writing – review and editing (supporting). J.J.P., methodology (supporting), investigation (supporting), and writing – review and editing (supporting). J.D.L., methodology (supporting) and writing – review and editing (supporting). D.A.D., conceptualization (lead), funding acquisition (lead), methodology (equal), project administration (lead), supervision (lead), methodology (supporting), visualization (supporting), writing – original draft (equal), and writing – review and editing (lead).

DECLARATION OF INTERESTS

D.A.D. serves as a member of the Scientific Advisory Board of Seawolf Therapeutics and has equity interest in the company. J.D.L. is a co-inventor of a technology used in this study and receives royalty payments related to the licensing of the technology from the University of Iowa. PCT/US2018/059065, filed November 2, 2018 (METHODS OF RESCUING STOP CODONS VIA GENETIC REASSIGNMENT WITH ACE-tRNA; Inventors, University of Iowa, J.D.L., and Christopher A. Ahern), pertains to the tRNA sequences used in this study.

SUPPLEMENTAL INFORMATION

Supplemental information can be found online at https://doi.org/10.1016/j.omtn.2025.102730.

REFERENCES

1. Dean, D.A. (1997). Import of plasmid DNA into the nucleus is sequence specific. Exp. Cell Res. *230*, 293–302.
2. Dean, D.A., Dean, B.S., Muller, S., and Smith, L.C. (1999). Sequence requirements for plasmid nuclear entry. Exp. Cell Res. *253*, 713–722.
3. Sebestyén, M.G., Ludtke, J.L., Bassik, M.C., Zhang, G., Budker, V., Lukhtanov, E.A., Hagstrom, J.E., and Wolff, J.A. (1998). DNA vector chemistry: the covalent attachment of signal peptides to plasmid DNA. Nature Biotech *16*, 80–85.
4. Swindle, C.S., Zou, N., Van Tine, B.A., Shaw, G.M., Engler, J.A., and Chow, L.T. (1999). Human papillomavirus DNA replication compartments in a transient DNA replication system. J. Virol. *73*, 1001–1009.
5. Tang, Q., Bell, P., Tegtmeyer, P., and Maul, G.G. (2000). Replication but not transcription of simian virus 40 DNA is dependent on nuclear domain 10. J. Virol. *74*, 9694–9700.
6. Ondrej, V., Kozubek, S., Lukásová, E., Falk, M., Matula, P., Matula, P., and Kozubek, M. (2006). Directional motion of foreign plasmid DNA to nuclear HP1 foci. Chromosome Res. *14*, 505–514.
7. Gasiorowski, J.Z., and Dean, D.A. (2007). Intranuclear Trafficking of Episomal DNA Is Transcription-dependent. Mol. Ther. *15*, 2132–2139.
8. Kopp, K., Gasiorowski, J.Z., Chen, D., Gilmore, R., Norton, J.T., Wang, C., Leary, D. J., Chan, E.K.L., Dean, D.A., and Huang, S. (2007). Pol I transcription and pre-rRNA processing are coordinated in a transcription-dependent manner in mammalian cells. Mol. Biol. Cell *18*, 394–403.
9. Larkin, J.D., Papantonis, A., and Cook, P.R. (2013). Promoter type influences transcriptional topography by targeting genes to distinct nucleoplasmic sites. J. Cell Sci. *126*, 2052–2059.
10. Nora, E.P., Lajoie, B.R., Schulz, E.G., Giorgetti, L., Okamoto, I., Servant, N., Piolot, T., van Berkum, N.L., Meisig, J., Sedat, J., et al. (2012). Spatial partitioning of the regulatory landscape of the X-inactivation centre. Nature *485*, 381–385.
11. Lieberman-Aiden, E., van Berkum, N.L., Williams, L., Imakaev, M., Ragoczy, T., Telling, A., Amit, I., Lajoie, B.R., Sabo, P.J., Dorschner, M.O., et al. (2009). Comprehensive mapping of long-range interactions reveals folding principles of the human genome. Science *326*, 289–293.
12. Chuang, C.H., Carpenter, A.E., Fuchsova, B., Johnson, T., de Lanerolle, P., and Belmont, A.S. (2006). Long-range directional movement of an interphase chromosome site. Curr. Biol. *16*, 825–831.
13. Tseng, W.C., Haselton, F.R., and Giorgio, T.D. (1997). Transfection by cationic liposomes using simultaneous single cell measurements of plasmid delivery and transgene expression. J. Biol. Chem. *272*, 25641–25647.
14. Cohen, R.N., van der Aa, M.A.E.M., Macaraeg, N., Lee, A.P., and Szoka, F.C., Jr. (2009). Quantification of plasmid DNA copies in the nucleus after lipoplex and polyplex transfection. J. Control. Release *135*, 166–174.
15. Wu, H., Qin, W., Lu, S., Wang, X., Zhang, J., Sun, T., Hu, X., Li, Y., Chen, Q., Wang, Y., et al. (2020). Long noncoding RNA ZFAS1 promoting small nucleolar RNA-mediated 2′-O-methylation via NOP58 recruitment in colorectal cancer. Mol. Cancer *19*, 95.
16. Lueck, J.D., Yoon, J.S., Perales-Puchalt, A., Mackey, A.L., Infield, D.T., Behlke, M.A., Pope, M.R., Weiner, D.B., Skach, W.R., McCray, P.B., Jr., and Ahern, C.A. (2019). Engineered transfer RNAs for suppression of premature termination codons. Nat. Commun. *10*, 822.
17. Hernandez-Verdun, D., Roussel, P., Thiry, M., Sirri, V., and Lafontaine, D.L.J. (2010). The nucleolus: structure/function relationship in RNA metabolism. Wiley Interdiscip. Rev. RNA *1*, 415–431.
18. Makinen, P.I., Koponen, J.K., Karkkainen, A.M., Malm, T.M., Pulkkinen, K.H., Koistinaho, J., Turunen, M.P., and Yla-Herttuala, S. (2006). Stable RNA interference: comparison of U6 and H1 promoters in endothelial cells and in mouse brain. J. Gene Med. *8*, 433–441.
19. Wu, L., Pan, J., Thoroddsen, V., Wysong, D.R., Blackman, R.K., Bulawa, C.E., Gould, A.E., Ocain, T.D., Dick, L.R., Errada, P., et al. (2003). Novel small-molecule inhibitors of RNA polymerase III. Eukaryot. Cell *2*, 256–264.
20. Qin, J.Y., Zhang, L., Clift, K.L., Hulur, I., Xiang, A.P., Ren, B.Z., and Lahn, B.T. (2010). Systematic comparison of constitutive promoters and the doxycycline-inducible promoter. PLoS One *5*, e10611.
21. Xu, M., and Cook, P.R. (2008). Similar active genes cluster in specialized transcription factories. J. Cell Biol. *181*, 615–623.
22. Evdokimov, A.G., Pokross, M.E., Egorov, N.S., Zaraisky, A.G., Yampolsky, I.V., Merzlyak, E.M., Shkoporov, A.N., Sander, I., Lukyanov, K.A., and Chudakov, D. M. (2006). Structural basis for the fast maturation of Arthropoda green fluorescent protein. EMBO Rep. *7*, 1006–1012.
23. Wang, J., Gao, G., and Wang, D. (2024). Developing AAV-delivered nonsense suppressor tRNAs for neurological disorders. Neurotherapeutics *21*, e00391.
24. Kim, S.Y., Lee, J.H., Shin, H.S., Kang, H.J., and Kim, Y.S. (2002). The human elongation factor 1 alpha (EF-1 alpha) first intron highly enhances expression of foreign genes from the murine cytomegalovirus promoter. J. Biotechnol. *93*, 183–187.
25. Zheng, C., and Baum, B.J. (2014). All human EF1alpha promoters are not equal: markedly affect gene expression in constructs from different sources. Int. J. Med. Sci. *11*, 404–408.
26. Lee, C.P., Ko, A.M.S., Chiang, S.L., Lu, C.Y., Tsai, E.M., and Ko, Y.C. (2018). Regulatory elements in vectors containing the ctEF-1alpha first intron and double enhancers for an efficient recombinant protein expression system. Sci. Rep. *8*, 15396.
27. Yeung, E., Dy, A.J., Martin, K.B., Ng, A.H., Del Vecchio, D., Beck, J.L., Collins, J.J., and Murray, R.M. (2017). Biophysical Constraints Arising from Compositional Context in Synthetic Gene Networks. Cell Syst. *5*, 11–24.e12.
28. Huang, S. (2002). Building an efficient factory: where is pre-rRNA synthesized in the nucleolus? J. Cell Biol. *157*, 739–741.

29. Misteli, T., and Spector, D.L. (1998). The cellular organization of gene expression. Curr. Opin. Cell Biol. *10*, 323–331.

30. Rao, D.D., Vorhies, J.S., Senzer, N., and Nemunaitis, J. (2009). siRNA vs. shRNA: similarities and differences. Adv. Drug Deliv. Rev. *61*, 746–759.

31. Sheng, P., Flood, K.A., and Xie, M. (2020). Short Hairpin RNAs for Strand-Specific Small Interfering RNA Production. Front. Bioeng. Biotechnol. *8*, 940.

32. Badding, M.A., Lapek, J.D., Friedman, A.E., and Dean, D.A. (2013). Proteomic and functional analyses of protein-DNA complexes during gene transfer. Mol. Ther. *21*, 775–785.

33. Badding, M.A., Vaughan, E.E., and Dean, D.A. (2012). Transcription factor plasmid binding modulates microtubule interactions and intracellular trafficking during gene transfer. Gene Ther. *19*, 338–346.

34. Ranganathan, V., Wahlin, K., Maruotti, J., and Zack, D.J. (2014). Expansion of the CRISPR-Cas9 genome targeting space through the use of H1 promoter-expressed guide RNAs. Nat. Commun. *5*, 4516.

35. Di Pascale, F., Nama, S., Muhuri, M., Quah, S., Ismail, H.M., Chan, X.H.D., Sundaram, G.M., Ramalingam, R., Burke, B., and Sampath, P. (2018). C/EBPbeta mediates RNA polymerase III-driven transcription of oncomiR-138 in malignant gliomas. Nucleic Acids Res. *46*, 336–349.

OMTN, Volume *36*

Supplemental information

Subnuclear organization and mislocalization of plasmids reduce transgene expression

Ningyang Gu, Uday K. Baliga, Joseph J. Porter, John D. Lueck, and David A. Dean

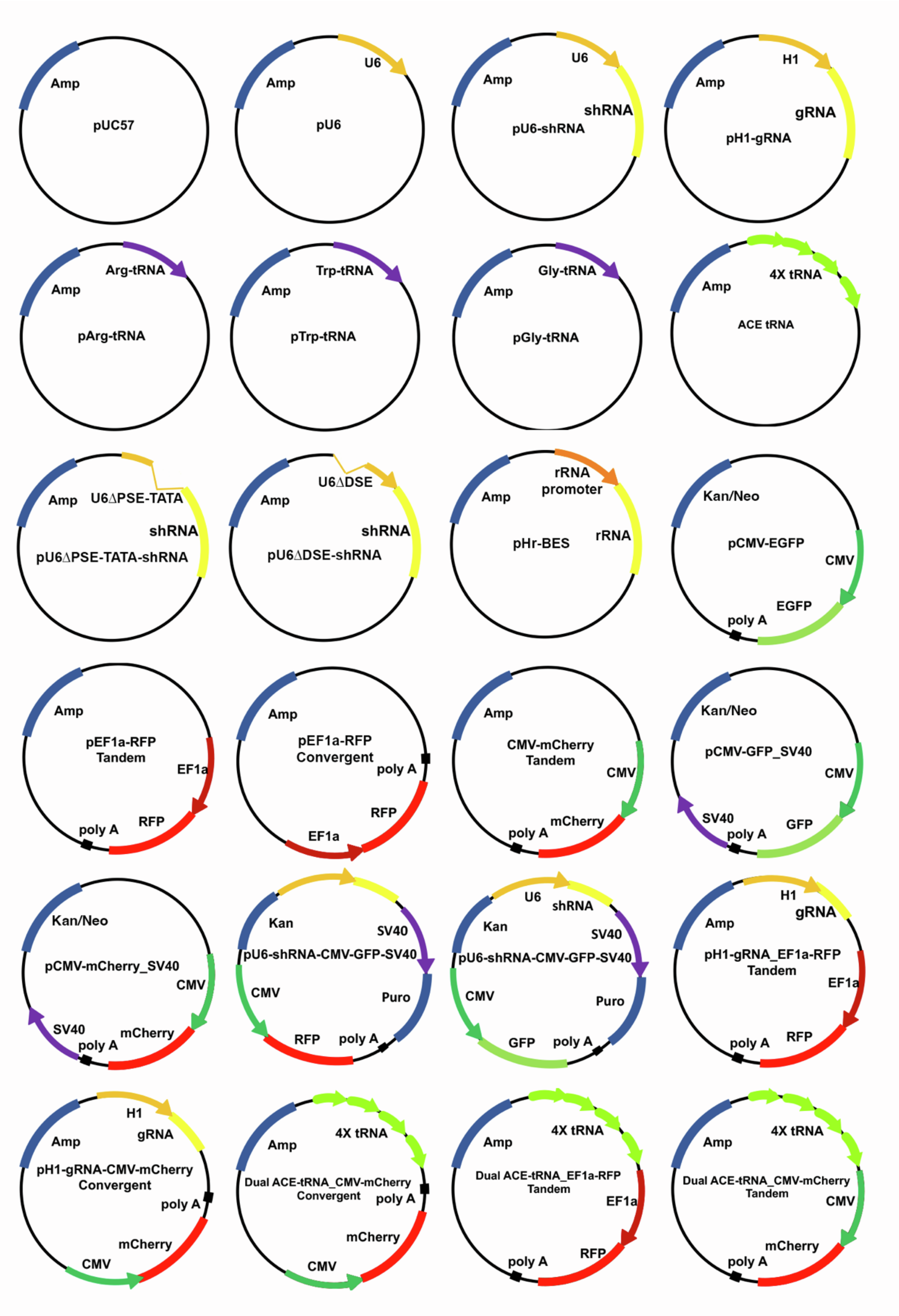

Figure S1. Plasmid Maps

Table S1. Effects of RNA polymerase inhibitors on endogenous Pol II and Pol III expression.

A549 cells were untreated (control), treated with actinomycin D (1 µg/ml) and α-amanitin (5 µg/ml) for 4 hours, or treated with ML-60218 (66 µM) for 24 hours prior to isolation of total RNA. Following reverse transcription, levels of U6 and c-myc were determined by qPCR using the ΔCt/ΔCt method. Primers 5'- CCTGGTGCTCCATGAGGAGAC-3' (c-myc forward), 5'-CAGACTCTGACCTTTTGCCAGG-3' (c-myc reverse), 5'- CGCTTCGGCAGCACATATAC-3' (U6 forward), and 5'- AAAATATGGAACGCTTCACGA-3' (U6 reverse) were used for amplification. Treatments were carried out using n=3-4 wells per condition.

Treatment	c-myc	U6
Control	1.041 ± 0.345	1.007 ± 0.286
1 µg/ml Actinomycin D + 5 µg/ml α-amanitin	0.008 ± 0.004	1.263 ± 0.258
66 µM ML-60218	0.969 ± 0.476	0.633 ± 0.021
